# Pt-Ru bimetallic nanoclusters with peroxidase-like activity for antibacterial therapy

**DOI:** 10.1371/journal.pone.0301358

**Published:** 2024-05-21

**Authors:** Chuang Wei, Yijun Gao, Peifeng Li

**Affiliations:** 1 Institute for Translational Medicine, College of Medicine, Qingdao University, Qingdao, China; 2 School of Medicine, Shanghai University, Shanghai, China; Fraunhofer USA, Inc. Center Midwest, UNITED STATES

## Abstract

Drug-resistant bacteria arising from antibiotic abuse infections have always been a serious threat to human health. Killing bacteria with toxic reactive oxygen species (ROS) is an ideal antibacterial method for treating drug-resistant bacterial infections. Here, we prepared Pt-Ru bimetallic nanoclusters (Pt-Ru NCs) with higher peroxidase (POD)-like activity than Pt monometallic nanoclusters. Pt-Ru can easily catalyze the decomposition of H_2_O_2_ to produce ·OH, thereby catalyzing the transformation of 3,3’,5,5’-tetramethylbiphenylamine (TMB) to blue oxidized TMB (oxTMB). We utilized the POD-like activity of the Pt-Ru NCs for antibacterial therapy. The results showed that at doses of 40 μg/mL and 16 μg/mL, the Pt-Ru NCs exhibited extraordinary antibacterial activity against *E*. *coli* and *S*. *aureus*, demonstrating the enormous potential of Pt-Ru NCs as antibacterial agents.

## 1. Introduction

Bacterial infections, especially drug-resistant bacterial infections caused by antibiotic abuse, has become an increasingly serious health threat worldwide [1, 2]. Bacteria exist in various corners of human skin and do not cause infection under normal circumstances [3, 4]. When the skin and mucous barrier are damaged, they invade, grow, reproduce, and secrete toxins, which gradually leads to the formation of acute/chronic infectious wounds over time [5, 6]. Furthermore, the widespread use of antibiotics has led to drug resistance issues worldwide, posing significant challenges to the treatment of bacterial infections in recent decades [7, 8]. Therefore, there is an urgent need to replace traditional treatment methods to combat bacterial infections.

In recent years, nanomaterials that mimic natural enzyme activity, known as nanozymes, have become a new strategy for combating microbial infections [9, 10]. Nanozymes are nanomaterials that replace natural enzymes by simulating the coordination environment of catalytic sites [11–17]. Due to their broad-spectrum antibacterial activity, low drug resistance, and high stability, nanozymes have many excellent properties [18, 19]. Through the sterilization mechanism of catalyzing the production of reactive oxygen species (ROS), the production of drug-resistant bacteria can be avoided in the treatment of bacterium-infected wounds [20, 21]. At present, the antibacterial mechanism of nanozymes is mainly believed to involve the production of highly toxic ROS, such as hydroxyl radicals (·OH), hydrogen peroxide (H_2_O_2_), superoxide anions (O^2–^), and singlet oxygen (^1^O_2_), through their own enzyme activities, such as catalase (CAT), oxidase (OXD), peroxidase (POD), superoxide dismutase (SOD), glutathione peroxidase, and glucose oxidase [22–24]. Oxidative stress reactions occur when the ROS produced are in contact with bacteria, resulting in lipid membrane peroxidation, which causes irreversible damage to the integrity of microbial cell membranes. The substances in the cells of microorganisms leak and decompose into small molecules such as H_2_O and CO_2_, which eventually leads to the complete inactivation of microorganisms [25, 26]. Compared with natural enzymes, nanozymes are more ideal antibacterial materials. Natural enzymes are susceptible to inherent protein characteristics and some inherent defects, such as low stability, complex production parameters, high cost, and potential immunogenicity, which limit their use in combating human infectious diseases [27, 28]. Nanozymes are nonimmunogenic, easy to generate, store, and transport and can operate under a wide range of conditions, such as pH, temperature, salt concentration, and redox microenvironment [29, 30]. Therefore, considering the acidity and high concentration of H_2_O_2_ in the bacterial internal environment, utilizing nanozymes for antibacterial treatment is a feasible alternative strategy.

**Fig 1:**
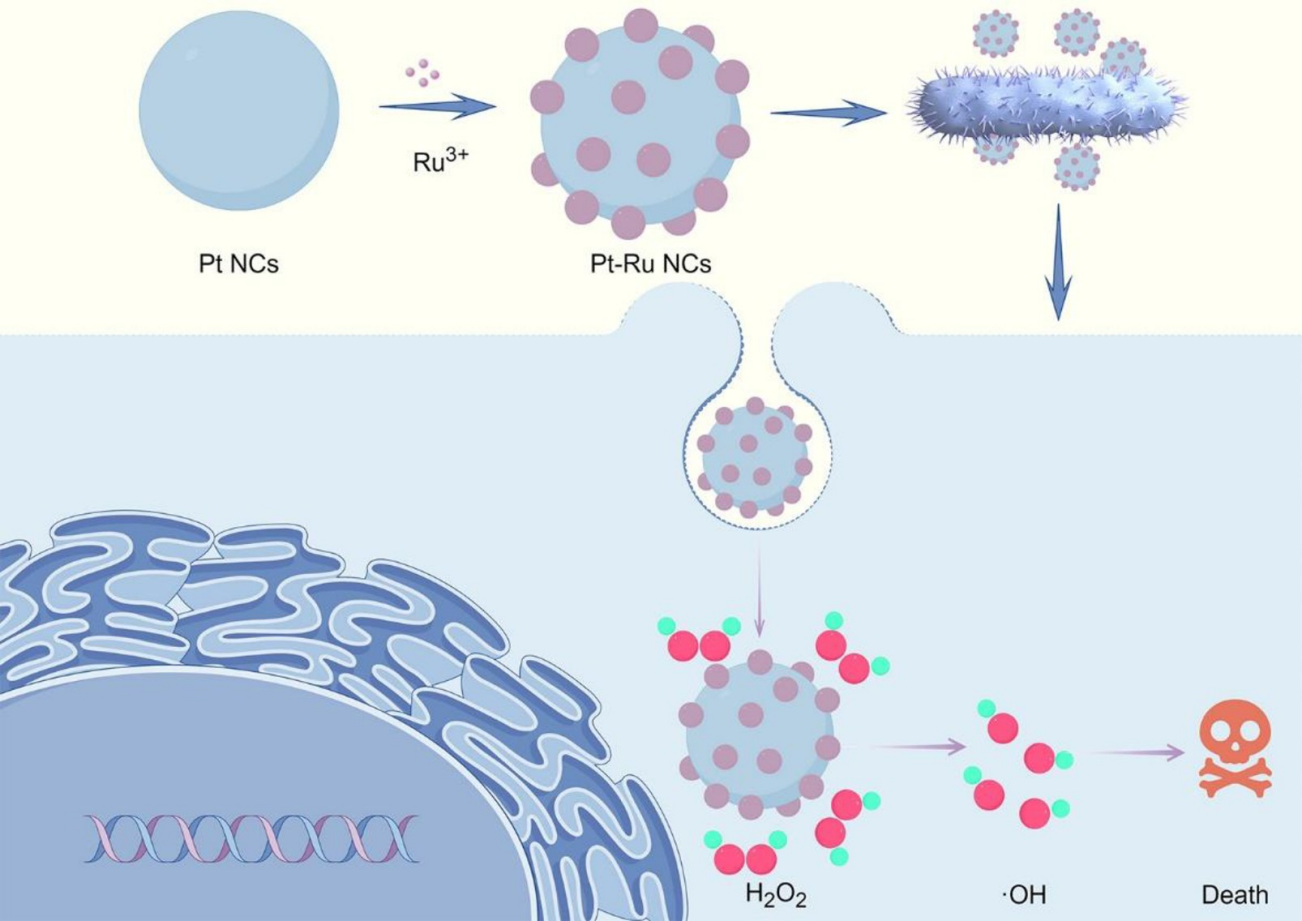
Synthesis and antibacterial schematic diagram of Pt-Ru NCs.

Here, we reported the simple synthesis and ultrahigh POD activity of bimetallic Pt-Ru nanoclusters (Pt-Ru NCs) and used them for antibacterial treatment (**Fig 1**). The results showed that Pt-Ru NCs showed extremely high catalytic efficiency for the substrates 3,3’,5,5’-tetramethylbiphenylamine (TMB) and H_2_O_2_, which was much greater than that of Pt monatomic nanozymes. In addition, we tested the activity of the Pt-Ru NCs at different pH values and temperatures, and the results showed that the activity of the Pt-Ru NCs showed excellent acid stability and temperature stability. Subsequent antibacterial tests showed that Pt-Ru NCs have excellent antibacterial properties even at low concentrations (40 μg/mL for *E*. *coli* and 16 μg/mL for *S*. *aureus*).

## 2. Materials and methods

### 2.1 Materials and reagents

Potassium tetrachloroplatinate(II) (K_2_PtCl_4_), ruthenium(III) chloride hydrate (RuCl_3_·xH_2_O), and 3,3′,5,5′-tetramethylbenzidine (TMB) were purchased from Aladdin (Shanghai, China). LB broth was purchased from Dalian Meilunbio Do., Ltd. Deionized water (with a specific resistance of 18.25 MΩ cm) was utilized for the preparation of all solutions throughout the experiments.

### 2.2. Instrument and characteristics

Transmission electron microscopy (TEM) images were obtained with a JEM-1400 instrument (JEOL). The hydrodynamic diameter was measured by dynamic light scattering (DLS) using a Malvern ZEN instrument. UV‒vis spectra were obtained via a UV‒vis spectrophotometer (Shimadzu, UV-1750).

### 2.3 Preparation of nanoclusters

The preparation of Pt-Ru NCs was based on previous reports with slight modifications [31]. Briefly, 40 mM K_2_PtCl_4_, 77 mM RuCl_3_, 12 mg glycine, 29 mg PVP and 2 mL ultrapure water were mixed and stirred for 10 min and then heated to 60°C for 5 min. 116 μM ascorbic acid was added to the mixture, and the mixture was incubated at 60°C for 2 h. Finally, the mixture was centrifuged and washed to obtain Pt-Ru NCs. In the same steps, RuCl3 was excluded to obtain Pt NCs.

### 2.4 Determination of catalytic performance

The POD-like activity of Pt-Ru NCs or Pt NCs was evaluated by determining the extent to which they catalyze the oxidation of TMB, and UV‒Vis absorption spectra from 500 nm to 800 nm were recorded. The absorption at 652 nm of the Pt-Ru NCs at different pH values (2, 4, 6, 8, 10) or temperatures (20, 25, 30, 35, 40°C) was detected using a UV‒visible spectrophotometer.

The steady-state dynamics analysis is based on previous research [35]. The calculation formula was as follows: b_nanozyme_ = *V* / (*ε* × *l*) × (Δ*A* / Δ*t*). SA = b_nanozyme_ / [m] = *V* / (*ε* × *l* × [*m*]) × (*ΔA / Δt*). *V* is the total volume of reaction solution (μL); *ε* is the molar absorption coefficient of the colorimetric substrate*; l* is the path length of light traveling in the cuvette (cm); and *ΔA/Δt* is the initial rate of change in absorbance at 652 nm min^−1^. SA = b_nanozyme_ / m. b_nanozyme_: Nanozyme catalytic activity (units). m is the nanozyme weight (mg). The Michaelis‒Menten equation and Lineaweaver-Burk equation are as follows: *V* = (*V*_max_ × *S*) / (*K*_m_ + *S*). *V*: initial reaction velocity; *V*_max_: maximal reaction rate; *S*: TMB concentration; *K*_m_: Michaelis constant.

### 2.5 Determination of the CAT and SOD-like activities of the nanozymes

The CAT and SOD-like activities of the nanozymes were measured using catalase activity detection kits (Solarbio, China) and superoxide dismutase detection kits (Solarbio, China), respectively.

### 2.6 Evaluation of antibacterial performance

In the presence of H_2_O_2_ (1.5 mM), different concentrations (0, 5, 10, 20 and 40 μg/mL) of Pt-Ru NCs were added to the same amount of *E*. *coli* suspension. Then, the suspension was incubated overnight at 37°C. The concentration of *E*. *coli* was measured at 600 nm using an enzyme-linked immunosorbent assay. The bacterial suspension was diluted and cultured on an agar plate for 12 h, after which the number of colonies was counted. The agar plate was prepared by solidifying a PBS solution containing 0.025 g/mL LB broth agar medium. The same steps were used to treat *S*. *aureus*, but the concentration of the Pt-Ru NCs was changed to 0, 2, 4, 8, or 16 μg/mL. Afterward, the antibacterial properties of the Pt-Ru NCs and Pt NCs were compared at the optimal antibacterial concentration.

### 2.7 Biological safety evaluation

Normal human renal epithelial HK2 cells were cultured in a cell culture incubator. After the cells were treated with nanozymes, cell viability was determined using a CCK-8 assay kit.

### 2.8 Statistical analysis

The obtained data are presented as the mean ± standard deviation (SD). The statistical significance was set at p < 0.05.

## 3. Results and discussion

### 3.1 Synthesis and characterization of Pt-Ru NCs

**Fig 2:**
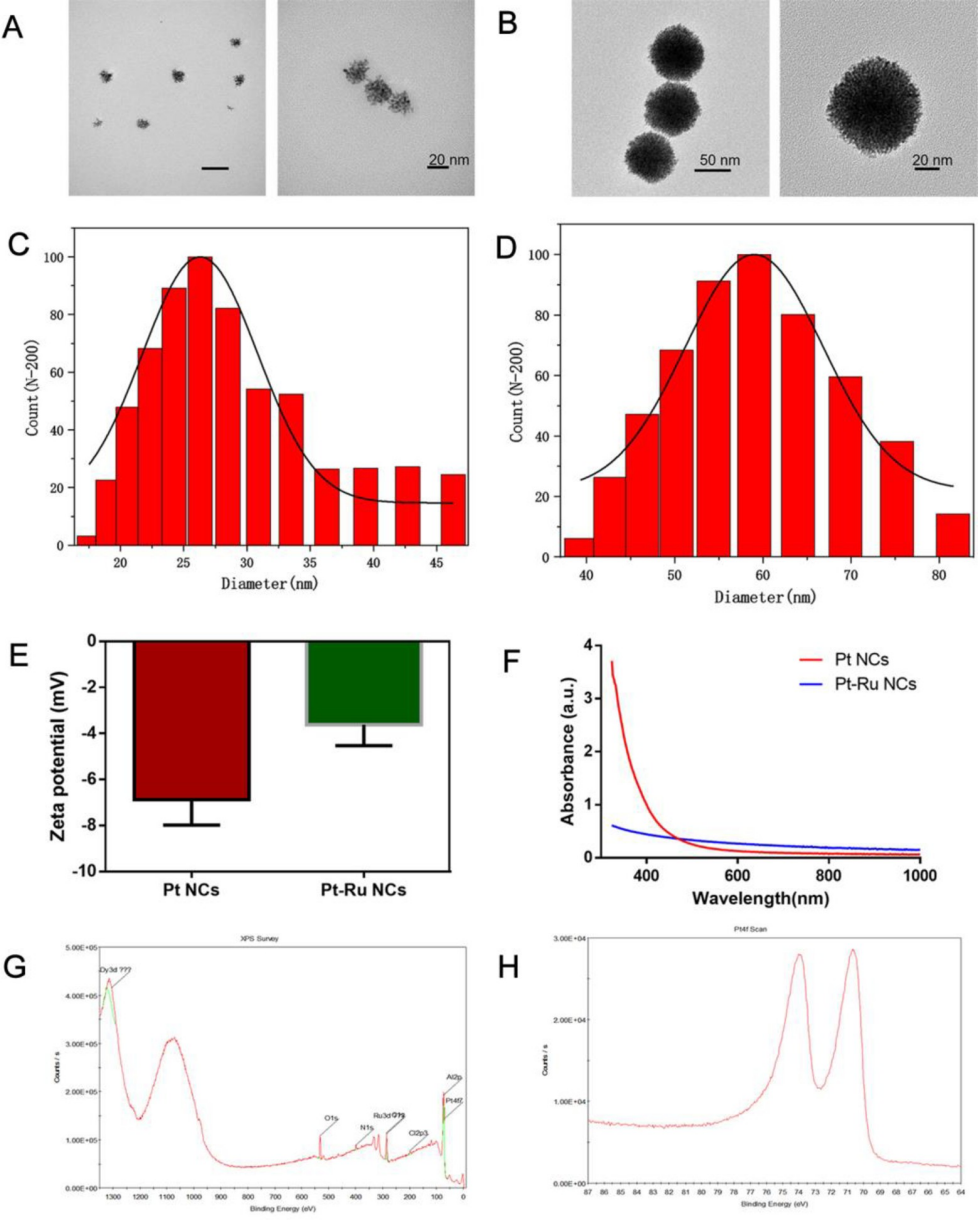
Characterization of nanoclusters.

First, Pt-Ru NCs were synthesized through a simple one-pot hydrothermal method. According to previous reports, Pt^2+^ can be directly reduced to Pt at 0.755 V, while Ru^3+^ requires two steps of reduction to Ru at 0.25 V (Ru^3+^to Ru^2+^) and 0.45 V (Ru^2+^to Ru) [32–35]. Therefore, ascorbic acid is used as the reducing agent, and Pt^2+^ is deposited as the first reduced object to form subparticles; then, Ru^3+^ is deposited on the Pt surface to form the final bimetallic Pt-Ru NCs. The morphologies of the prepared NCs were detected by TEM, and the hydrodynamic diameter was measured by DLS. As shown in **Fig 2A**, the prepared Pt exhibited nanoclusters with a particle size of approximately 27 nm (**Fig 2B**). Due to the deposition of Ru, the prepared Pt-Ru not only presented a nanocluster shape (**Fig 2C**) but also had a large particle size of approximately 60 nm (**Fig 2D**). The subsequent potential test showed that the potential of the Pt-Ru NCs was slightly greater than that of the Pt NCs (–5 vs. –8 mV) (**Fig 2E**). The UV‒Vis absorption results of Pt NCs and Pt-Ru NCs show that compared to the high absorption of Pt NCs at 0–400 nm, the deposition of Ru leads to a rapid decrease in the absorption of Pt-Ru NCs (**Fig 2F**), which can be explained by the fact that due to different atomic radii, the deposition of Ru on the subparticle surface disrupts the ordered growth of Pt seeds. XPS analysis revealed characteristic signals corresponding to N 1 s, O 1 s, Pt 4f, and Ru 3d in the Pt-Ru NCs, indicating the presence of not only Pt and PVP but also the successful doping of Ru into bimetallic NCs (**Fig 2G**). The binding energies of Pt4f5/2 and Pt4f7/2 in the Pt-Ru NCs were 74 eV and 71 eV, respectively (**Fig 2H**). The above results confirm the successful preparation of Pt-Ru NCs. Furthermore, the addition of Ru atoms can accelerate the reduction rate of Pt^2+^, which is demonstrated by the decrease in the color of the NCs after different hydrothermal reduction times (**S1 Fig**). As a result, Pt-Ru NCs have a larger specific surface area and more catalytic active sites than Pt NCs, thus exhibiting extremely high POD-like activity.

### 3.2 Determination of POD-like activity

**Fig 3:**
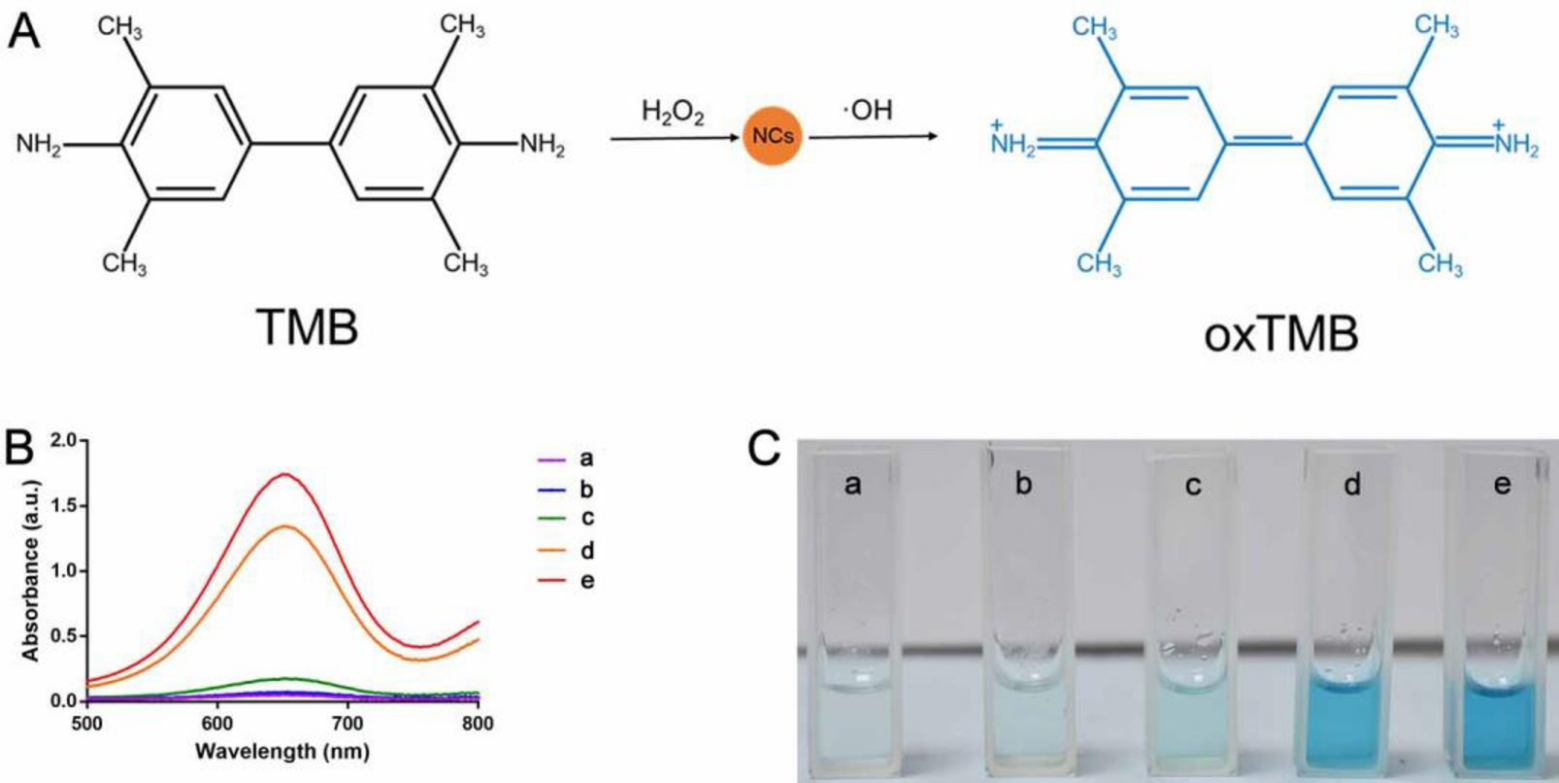
Identification of POD-like activity.

In the presence of H_2_O_2_, nanomaterials with POD-like activity can promote the decomposition of H_2_O_2_ to generate strongly oxidizing ·OH; thus, TMB can be oxidized to blue oxidized TMB (oxTMB), which exhibits characteristic absorbance at 652 nm (**Fig 3A**). Therefore, in this work, TMB was used as the catalytic substrate to evaluate the POD-like activity of Pt-Ru NCs or Pt NCs. As shown in **Fig 3B** and **3C**, negligible absorption and an approximately colorless solution were observed in the absence of nanoclusters, indicating that the mixture of H_2_O_2_ and TMB cannot mediate the generation of oxTMB. Similar results were also observed for the mixture of H_2_O_2_ and Pt-Ru NCs, indicating that the lack of TMB cannot mediate the generation of oxTMB. A mild absorption and light blue solution were observed for the TMB + Pt-Ru NCs, indicating that Pt-Ru NCs can promote the transformation of TMB to oxTMB at a low rate. This result indicates that Pt-Ru has a slight oxidase activity. However, when Pt-Ru NCs or Pt NCs were added to the mixture of TMB and H_2_O_2_, a rapid increase in absorption and a clear blue color were observed, and the mixture with Pt-Ru NCs had greater absorption and a deeper blue color than did the mixture with Pt NCs; this indicates that both Pt-Ru NCs and Pt NCs can catalyze the conversion of TMB to oxTMB, and the POD-like activity of Pt-Ru NCs is greater than that of Pt NCs. In addition, we investigated whether Pt-Ru has CAT and SOD activities. The results indicate that Pt-Ru exhibits insignificant SOD (**S2A Fig**) and CAT-like activity (**S2B Fig**). These results confirm that Pt-Ru is primarily characterized by POD-like activity.

### 3.3 Determination of POD-like activity

**Fig 4:**
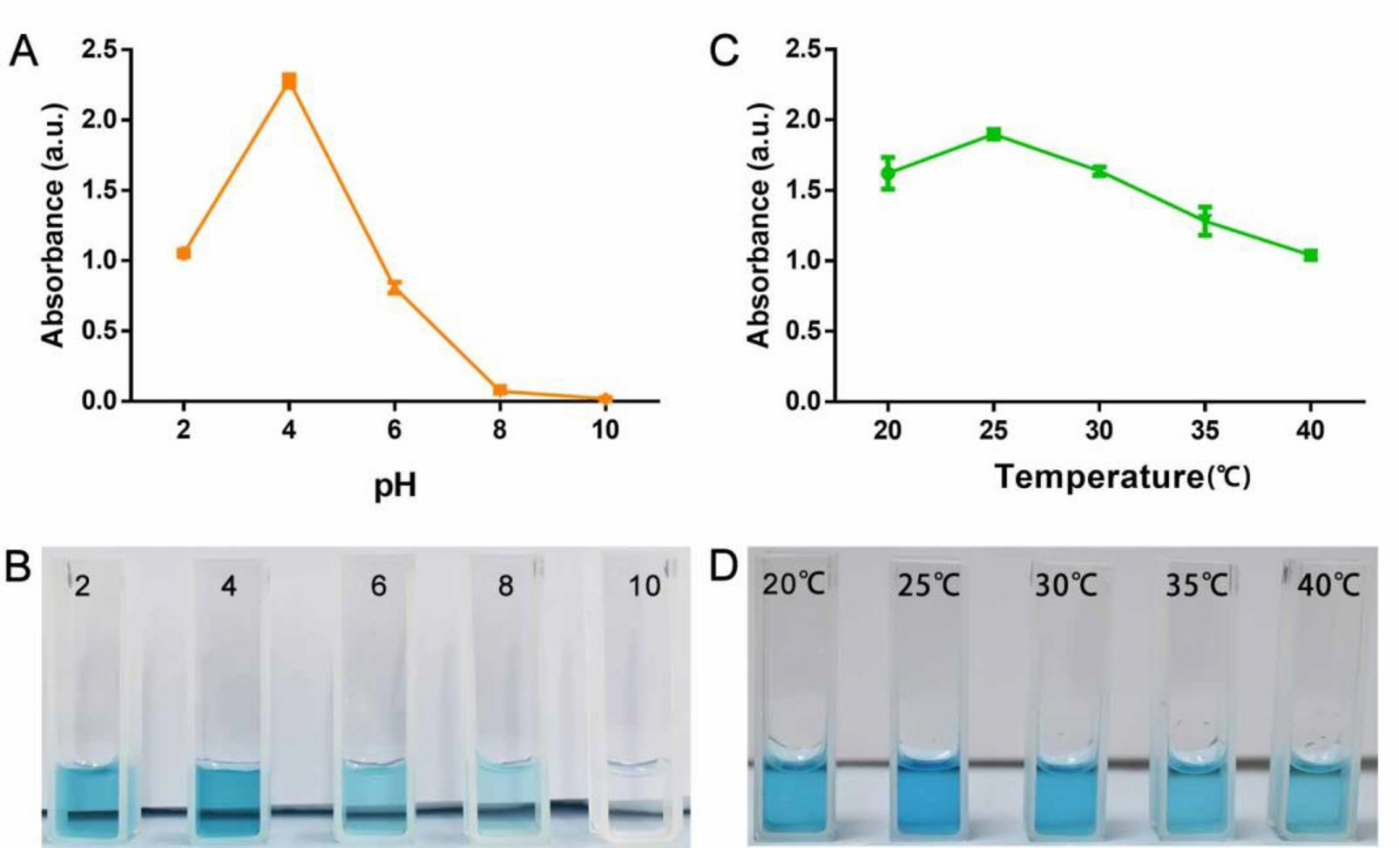
Stability identification of POD-like activity.

Considering that the suitable environment for bacterial growth is acidic and 37°C, we studied the stability of the POD-like activity of Pt-Ru NCs at different pH values (2, 4, 6, 8 and 10) and temperatures (20, 25, 30, 35 and 40°C). The results showed that, on the one hand, the Pt-Ru NCs exhibited stable activity in acidic environments, and the activity was strongest at pH = 4. As the pH increased, the activity of the Pt-Ru NCs gradually decreased and was almost completely lost in alkaline environments (**Fig 4A, 4B, S3 Fig**). On the other hand, the Pt-Ru NCs exhibited the strongest activity at 25°C and still exhibited excellent activity stability at 37°C (**Fig 4C, 4D and S4 Fig**). These results indicate that Pt-Ru NCs can maintain good activity in acidic and 37°C environments and have potential antibacterial applications.

### 3.4 Steady-state kinetic analysis

**Fig 5:**
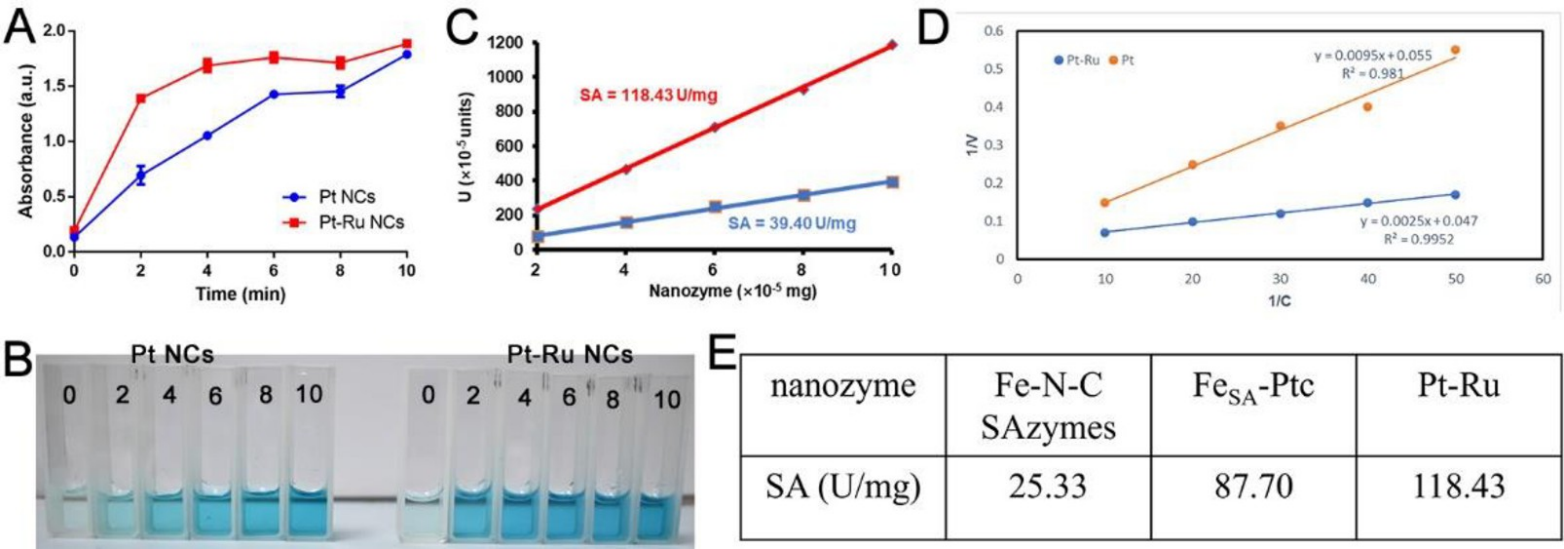
Steady-state kinetic analysis of Pt-Ru NCs.

Next, we conducted a kinetic analysis of nanoclusters through formula calculations to clarify their specific activity. First, the catalytic rates of the Pt-Ru NCs and Pt NCs were studied. The results showed that at the same time, the absorption of the mixture of H_2_O_2_ and TMB treated with Pt-Ru NCs increased sharply at 652 nm within 2 minutes and approached its maximum value at 4 minutes, while Pt NCs showed a slow increasing absorption curve and approached its maximum value at 10 minutes (**Fig 5A, 5B, S5 and S6 Figs**), indicating that the catalytic rate of Pt-Ru NCs was significantly greater than that of Pt NCs. Next, we conducted formula calculations, and the results showed that the SA of the Pt-Ru NCs was 118.43 U/mg, while the SA of the Pt NCs was 39.40 U/mg. The K_m_ and V_max_ of the Pt-Ru NCs were 0.05 and 18×10^−6^ M S^-1^, respectively. The K_m_ and V_max_ of the Pt NCs were 0.17 and 21×10^−6^ M S^-1^, respectively. These results confirmed that the nanozyme activity of the Pt-Ru NCs was significantly greater than that of the Pt NCs (**Fig 5C and 5D**). In addition, the excellent enzyme activity of Pt-Ru was further confirmed by comparing its POD-like activity with that of nanozymes reported in the literature (**Fig 5E**) [36, 37].

### 3.5 Detection of OH

Thiourea is considered an effective free radical scavenger. The results showed that a significant absorption peak was observed at 652 nm before the addition of thiourea, while negligible absorption peak was observed after the addition of thiourea (**S7A Fig**), indicating the generation of ·OH in the Pt-Ru + H_2_O_2_ + TMB reaction system. The fluorescent probe TA, which specifically labels ·OH, has also been used to detect the presence of ·OH. The results indicate that the fluorescence intensity is dependent on the concentration of Pt-Ru in the Pt-Ru + H_2_O_2_ + TMB reaction system (**S7B Fig**), indicating that the content of ·OH increases with increasing Pt-Ru concentration. These results confirm that Pt-Ru can catalyze the generation of ·OH to exert POD-like activity.

### 3.6 Evaluation of antibacterial performance

**Fig 6:**
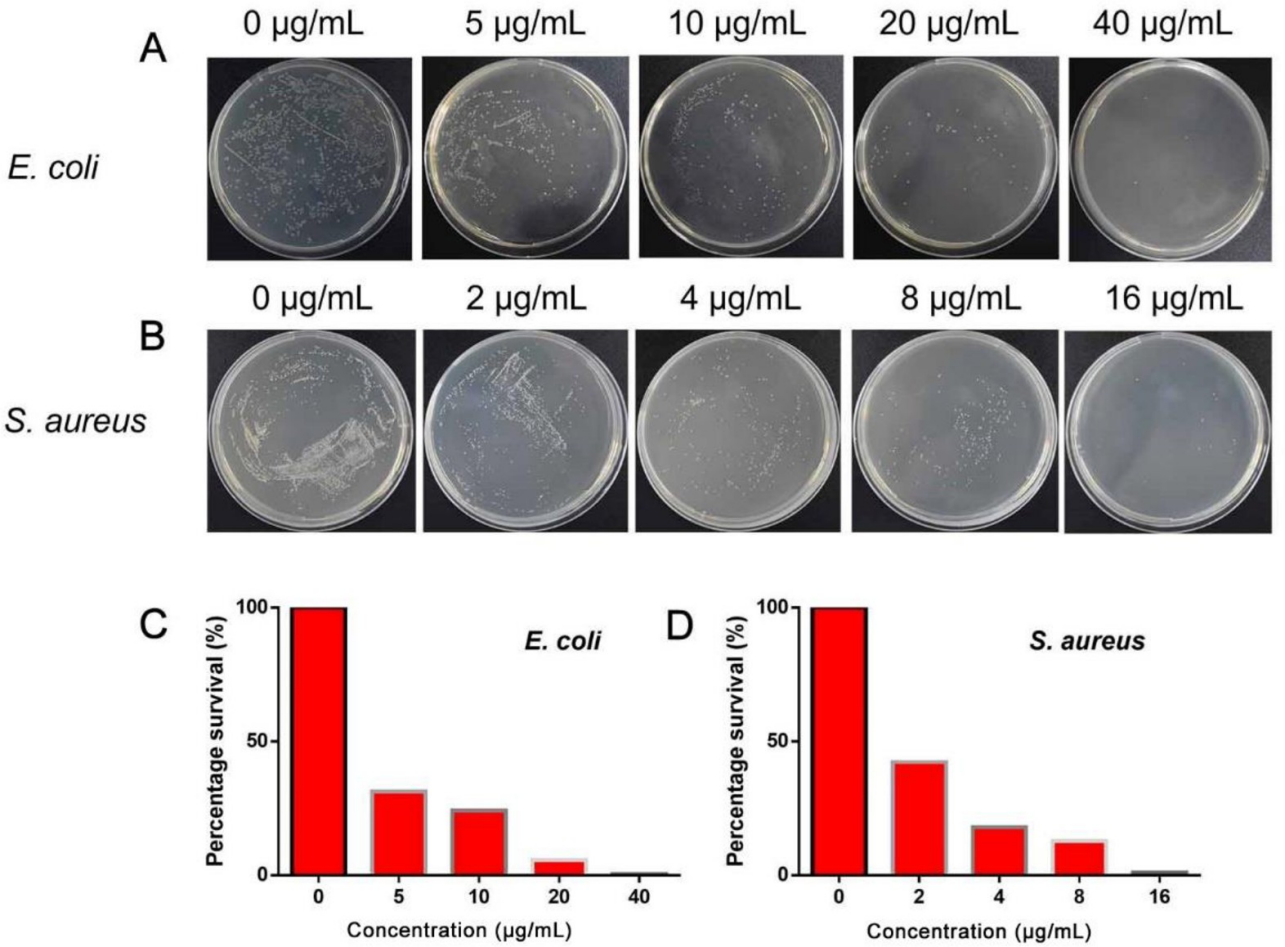
The antibacterial performance of Pt-Ru NCs in the presence of 1.5 mM H_2_O_2_.

To evaluate the antibacterial performance of the Pt-Ru NCs, we selected *E*. *coli* as the representative gram-negative bacteria and *S*. *aureus* as the representative gram-positive bacteria. First, the optimal inhibitory concentration of Pt-Ru NCs against two types of bacteria was detected. Bacterial coating experiments showed that as the concentration of Pt-Ru NCs gradually increased from 0 μg/mL to 40 μg/mL, the number of *E*. *coli* colonies gradually decreased (**Fig 6A**). The antibacterial activity of 40 μg/mL Pt-Ru NCs reached 99.63% (**Fig 6C**). Similarly, as the concentration of Pt-Ru NCs gradually increased from 0 to 16 μg/mL, the number of *S*. *aureus* colonies gradually decreased (**Fig 6B**). The antibacterial activity of 16 μg/mL Pt-Ru NCs reached 98.95% (**Fig 6D**). These results confirmed the good antibacterial ability of the Pt-Ru NCs.

**Fig 7:**
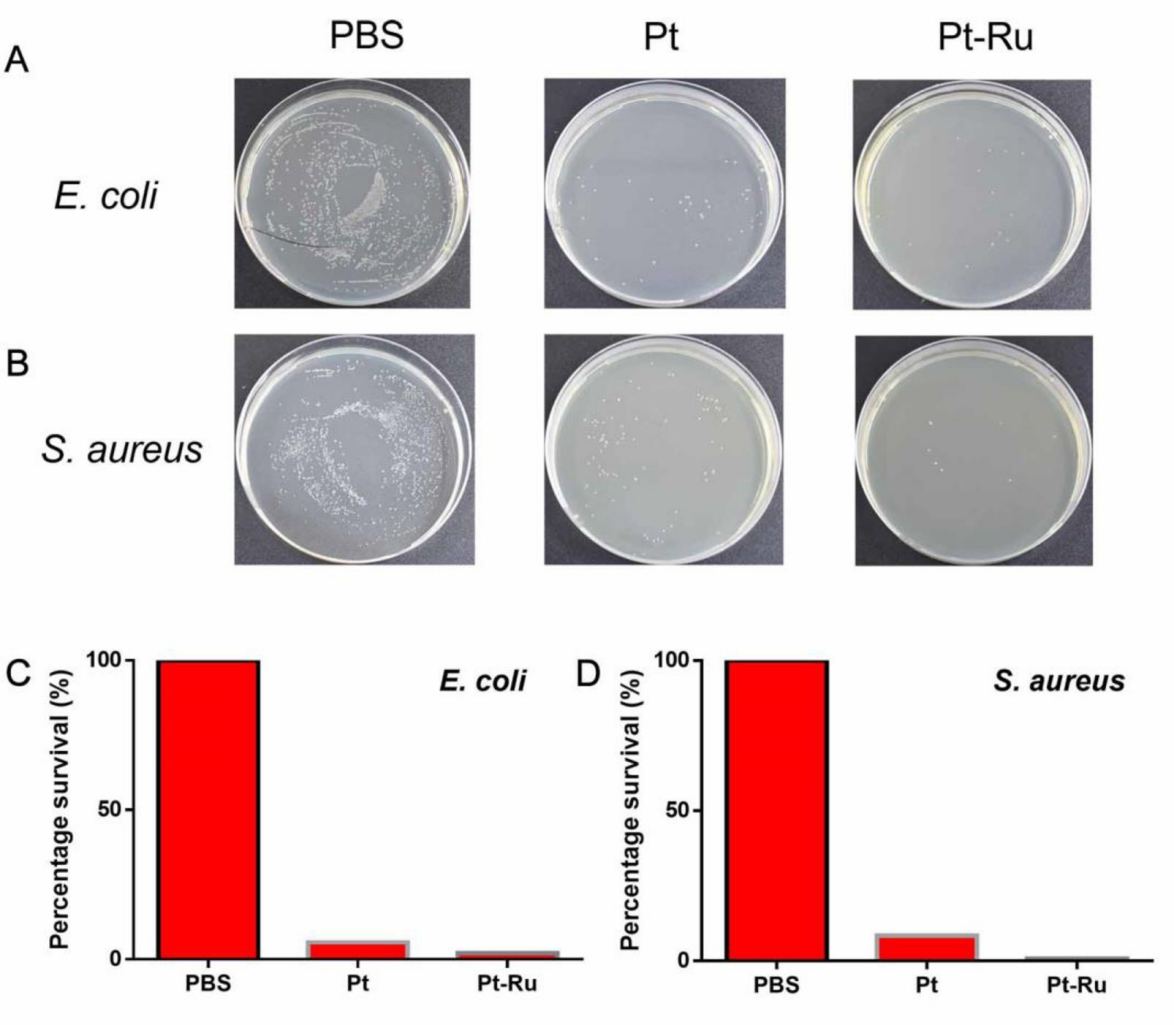
Comparison of antibacterial properties of different nanoclusters in the presence of 1.5 mM H_2_O_2_.

Next, the survival rates of bacteria subjected to different treatments at optimal antibacterial concentrations were compared. As shown in **Fig 7A and 7C**, with the PBS group as the reference control, the survival rate of *E*. *coli* in the Pt NC group was 5.71%, while the survival rate in the Pt-Ru NC-treated group was 2.05%, which was significantly lower than that in the Pt NC group. Similar results were also observed for *S*. *aureus*. The survival rate of the Pt NC group was 8.41%, while the survival rate of the Pt-Ru NC group was 0.77%, which was significantly lower than that of the Pt NC group (**Fig 7B and 7D**). These results show that the higher POD-like activity of Pt-Ru NCs compared to that of Pt NCs improves their antibacterial ability.

### 3.7 Anti-biofilm efficacy of Pt-Ru

To evaluate the effect of nanoclusters on bacterial biofilm formation, crystal violet staining was used to quantify biofilm formation. A bacterial solution concentration of 5 × 10^7^ CFU/mL was used to form the bacterial biofilms. After treatment with different nanoclusters, the biofilms stained with crystal violet were semiquantitatively analyzed. Compared to those in the PBS treatment group, the biofilms of *E*. *coli* in the Pt treatment group were slightly removed, while the biofilms of *E*. *coli* in the Pt-Ru treatment group were significantly removed (**S8A Fig**). A similar trend was observed for *S*. *aureus* (**S8B Fig**). These results further confirmed the excellent antibacterial effect of Pt-Ru, which disrupted the biofilm.

### 3.8 Comparison of antibacterial properties with those of other nanozymes

To better demonstrate the antibacterial performance of Pt-Ru, we utilized several nanozymes with POD-like activity reported in the literature for antibacterial therapy. The results showed that the colony count and catalytic activity trend of nanozymes at the same concentration after bacterial treatment were consistent (**S9 Fig**), indicating that Pt-Ru, which had the highest POD-like activity, also had the best antibacterial effect.

### 3.9 Biosafety evaluation of nanoclusters

The low toxicity of nanomaterials is highly important for future practical applications. To evaluate the biosafety of the nanoclusters, HK-2 cells were incubated with the nanoclusters, and cell viability was measured. **S10 Fig** shows that as the concentration of nanoclusters increased, the cell viability of the Pt-treated group and the Pt-Ru-treated group did not significantly decrease compared to that of the PBS-treated group. Even at a concentration of 200 μg/mL, HK-2 cells still maintained over 90% viability, indicating that both nanoclusters used in this study have good biosafety.

## 4. Conclusion

The increase in the prevalence of drug-resistant bacteria caused by antibiotic abuse is considered a global health threat. Fortunately, nanozymes with natural enzyme activity provide an option for preventing bacterial resistance. Therefore, our goal was to develop an effective nanozyme for antibacterial therapy. We prepared Pt-Ru bimetallic nanoclusters with higher POD-like activity than Pt monometallic nanozymes. After treating bacteria with Pt-Ru NCs, Pt-Ru NCs can catalyze the generation of toxic ·OH from H_2_O_2_ in the bacterial body, leading to bacterial death. There is still room for further improvement. First, we must further discuss the antibacterial effect of Pt-Ru NCs in animals. Second, due to their high surface energy, Pt-Ru NCs are not very stable and are prone to aggregation, which may hinder their clinical translation. Nevertheless, in this study we explored the application of Pt-Ru NCs in the field of antibacterial agents, which may contribute to providing solutions for the development of drug resistance in the future for the treatment of various microbial infections.

## Supporting information

S1 Fig The color changes of the solution corresponding to different hydrothermal times. Left: Pt NCs; Right: Pt-Ru NCs.
(TIF)

S2 Fig Identification of other enzyme-like activities of nanoclusters. (TIF)

S3 Fig The absorption curves after Pt-Ru NCs treatments in various pH solutions. (TIF)

S4 Fig The absorption curves after Pt-Ru NCs treatments in various temperature. (TIF)

S5 Fig The time-dependent absorption curves after Pt NCs treatments. (TIF)

S6 Fig The time-dependent absorption curves after Pt-Ru NCs treatments. (TIF)

S7 Fig Detection of ·OH. (TIF)

S8 Fig Evaluation of damage to bacterial biofilm by different treatments in the presence of 1.5 mM H_2_O_2_. (TIF)

S9 Fig Comparison of antibacterial properties between Pt-Ru and other nanozymes with POD-like activity. (TIF)

S10 Fig Evaluation of the safety capability of nanoclusters. (TIF)

S1 Raw data (RAR)
