# Supplementary figures and images for: Pt-Ru bimetallic nanoclusters with peroxidase-like activity for antibacterial therapy
Source: PLoS One. 2024 May 21;19(5):e0301358. doi: 10.1371/journal.pone.0301358 (PMC11108137; doi:10.1371/journal.pone.0301358)

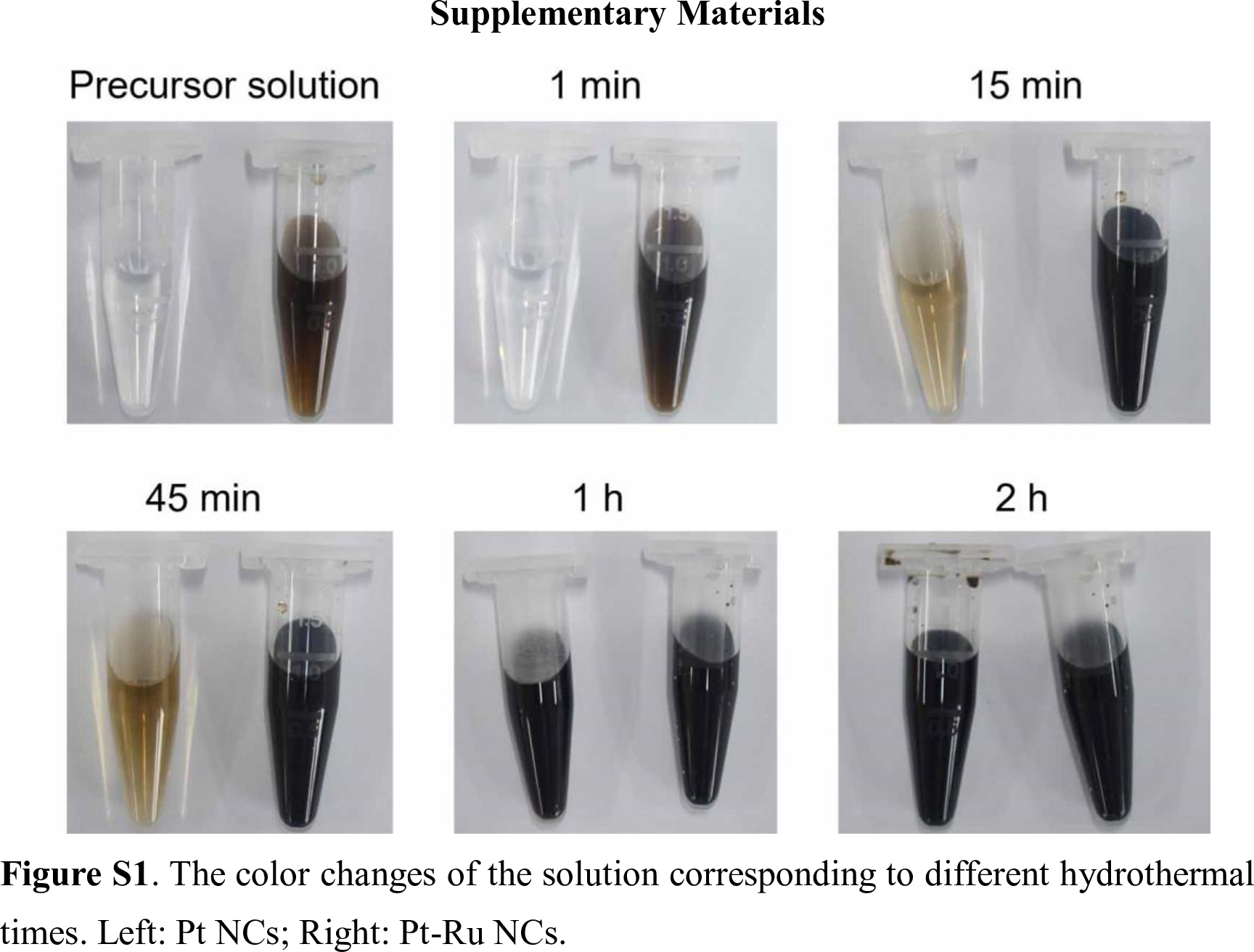

Supplement: S1 Fig — Left: Pt NCs; Right: Pt-Ru NCs. (TIF) [file pone.0301358.s001.tif]

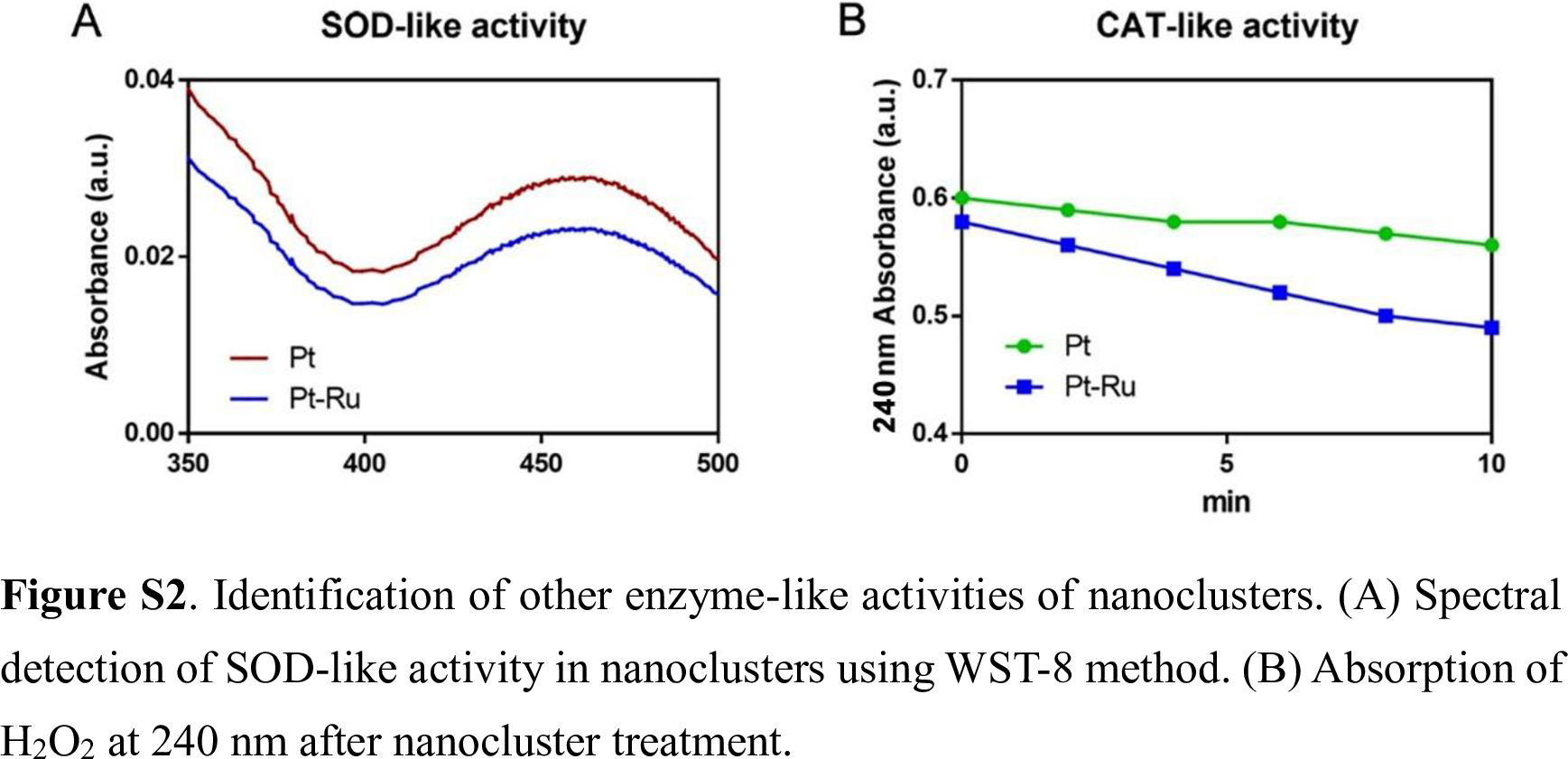

Supplement: S2 Fig — (TIF) [file pone.0301358.s002.tif]

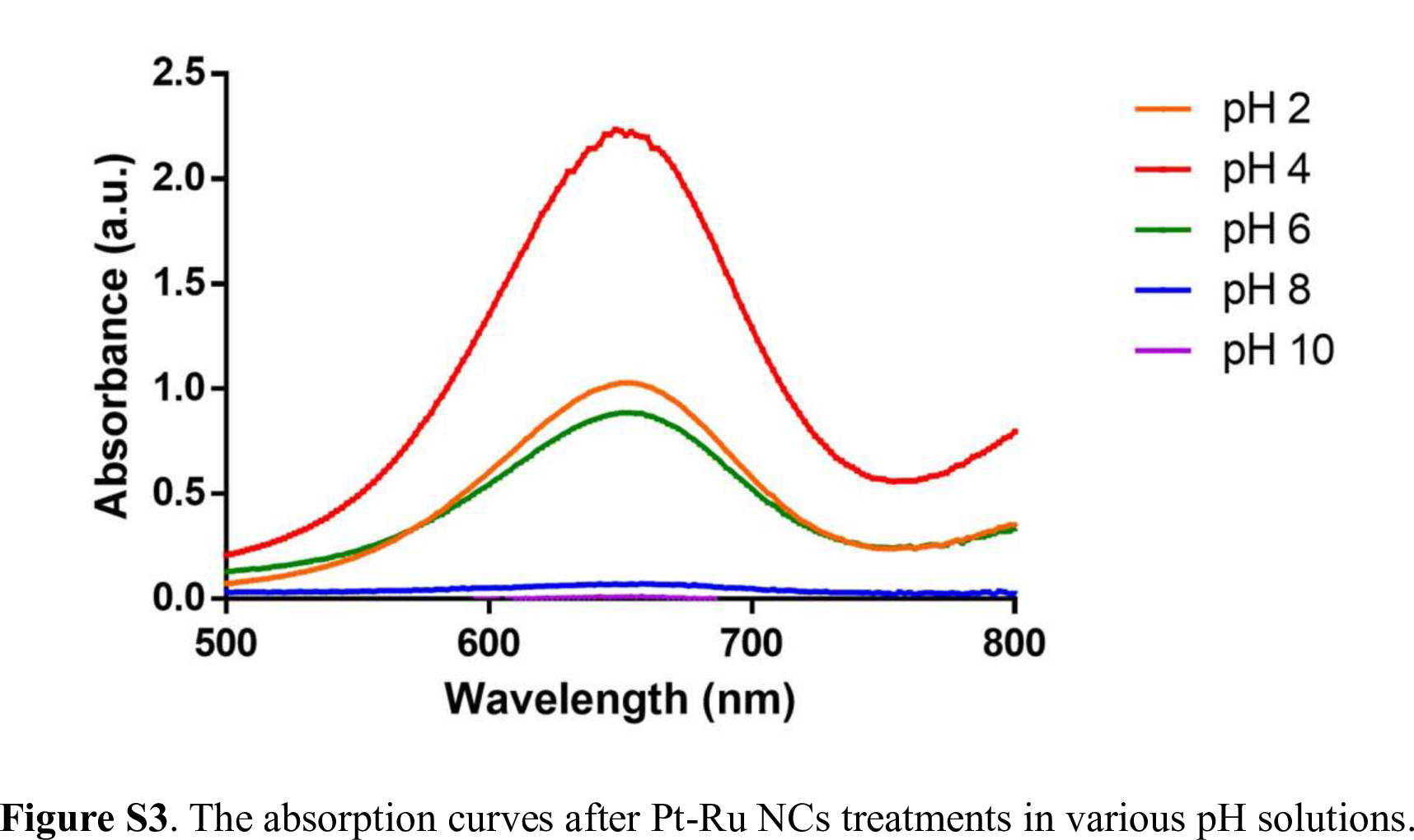

Supplement: S3 Fig — (TIF) [file pone.0301358.s003.tif]

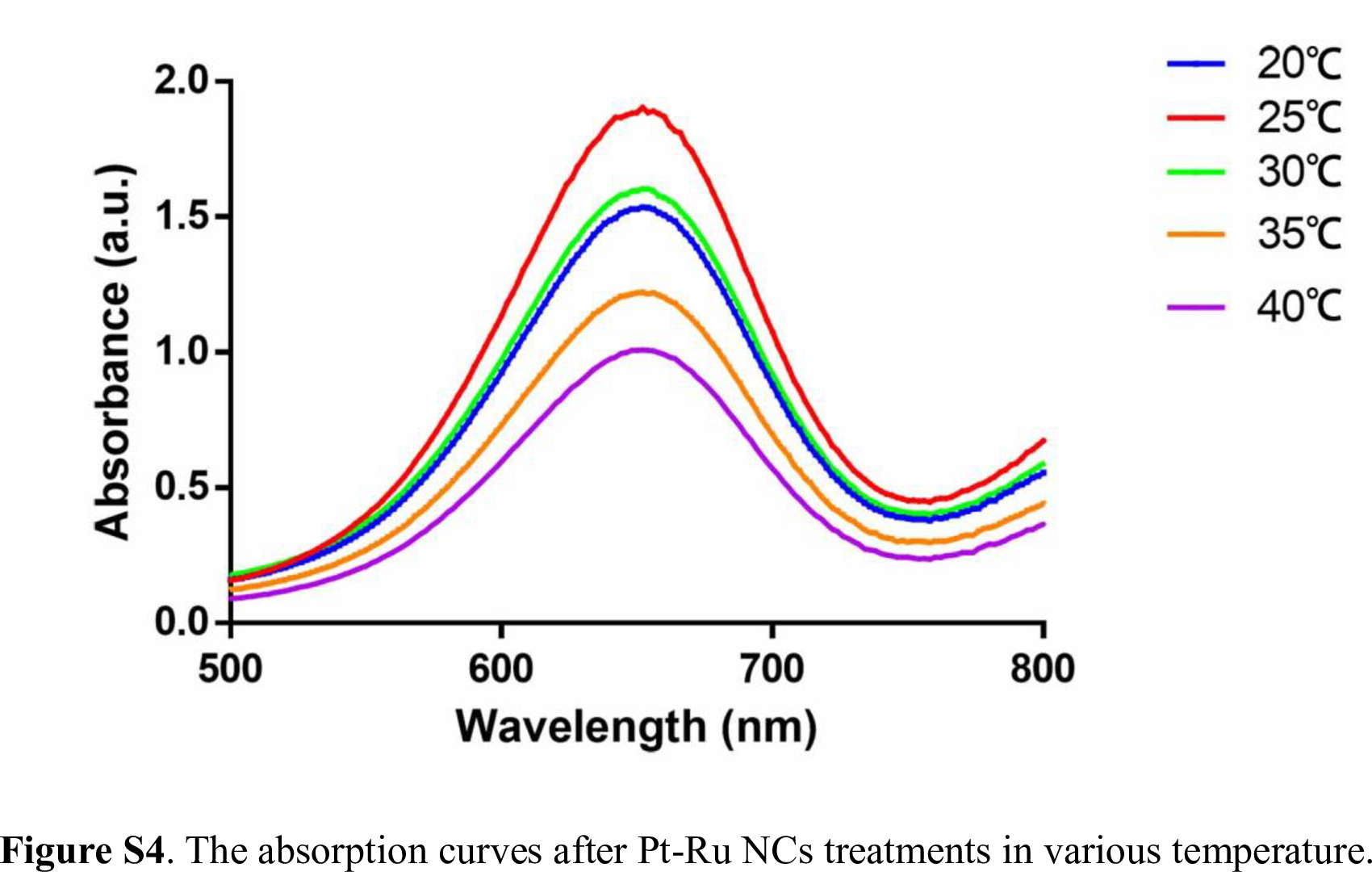

Supplement: S4 Fig — (TIF) [file pone.0301358.s004.tif]

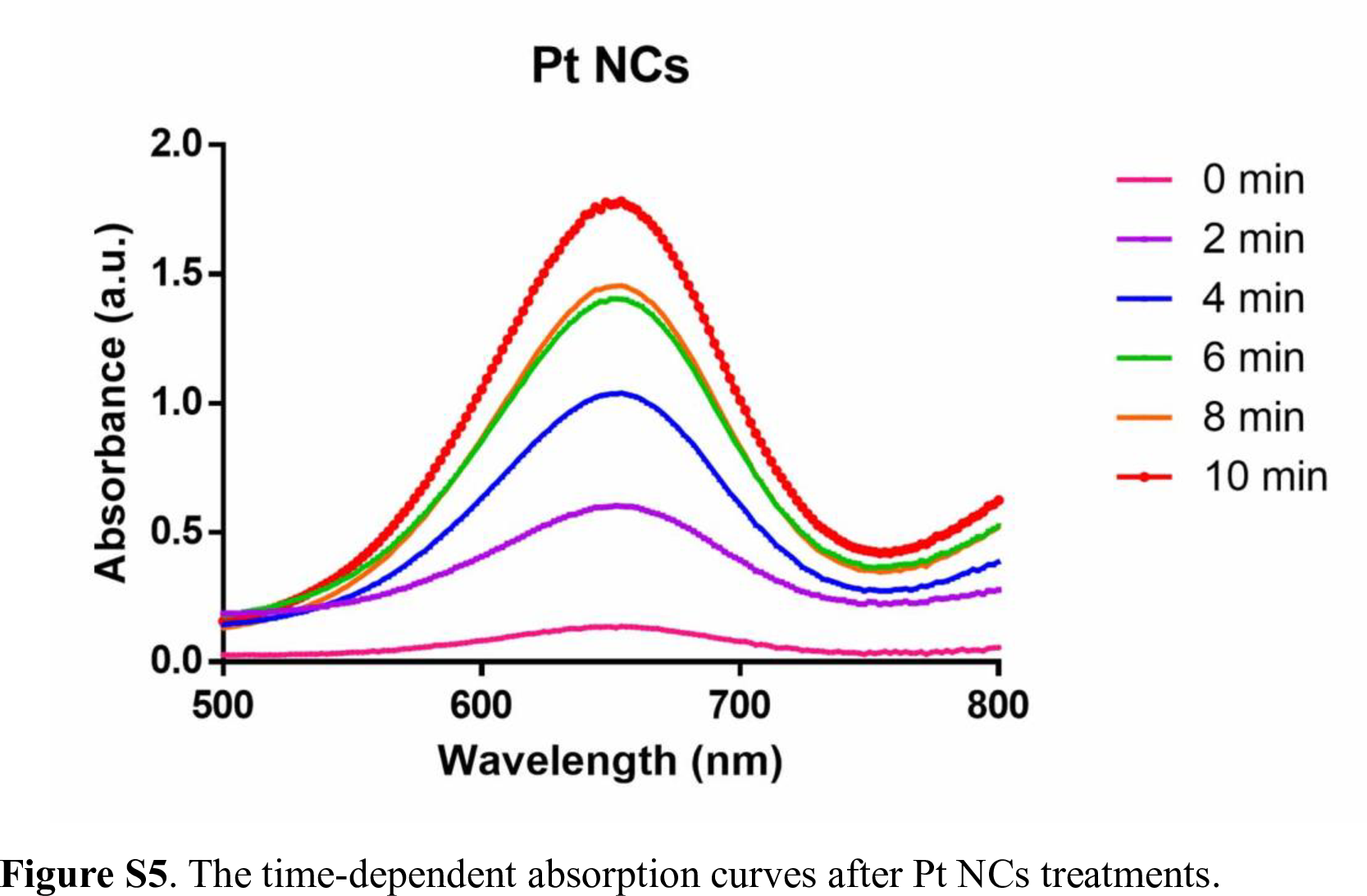

Supplement: S5 Fig — (TIF) [file pone.0301358.s005.tif]

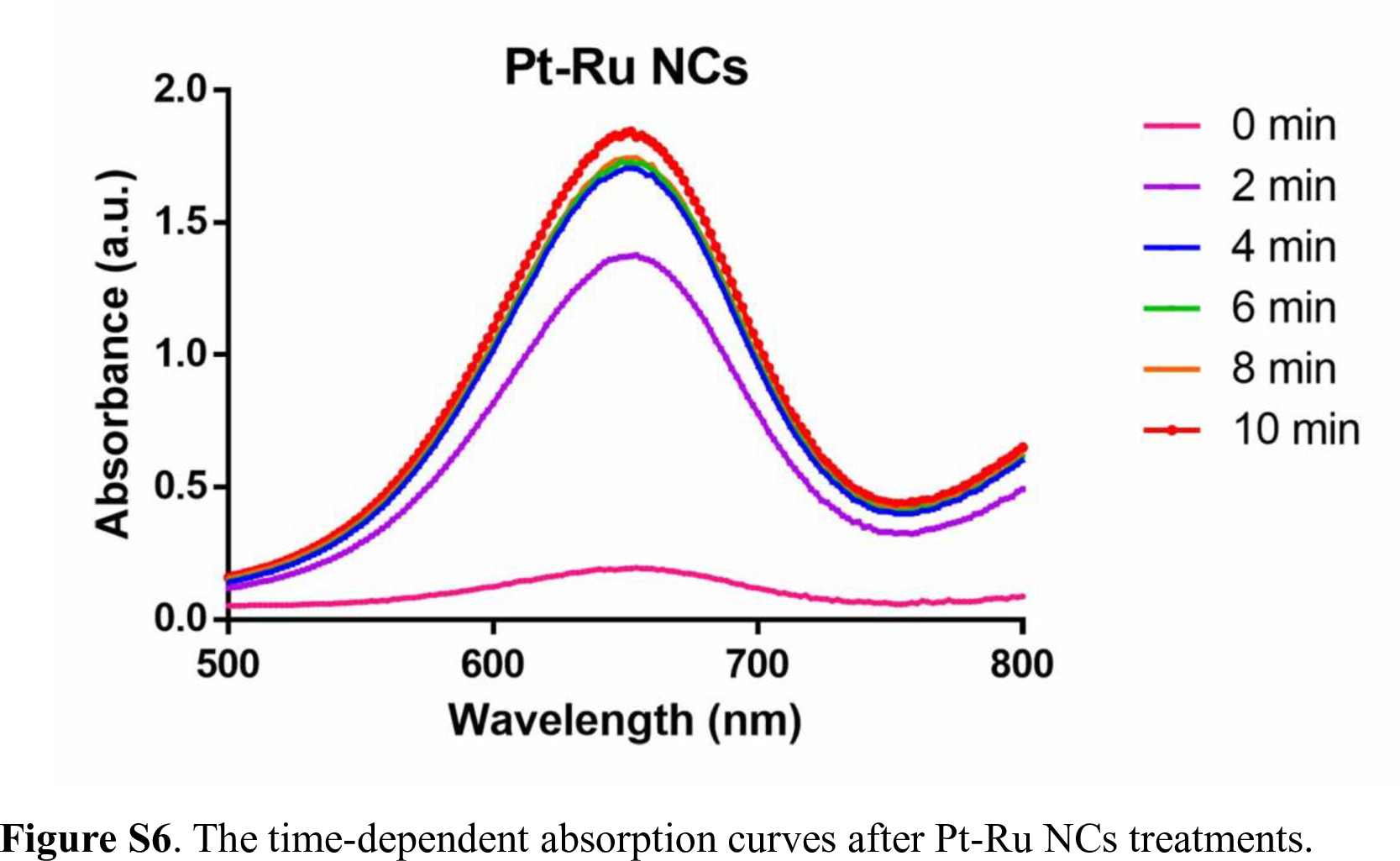

Supplement: S6 Fig — (TIF) [file pone.0301358.s006.tif]

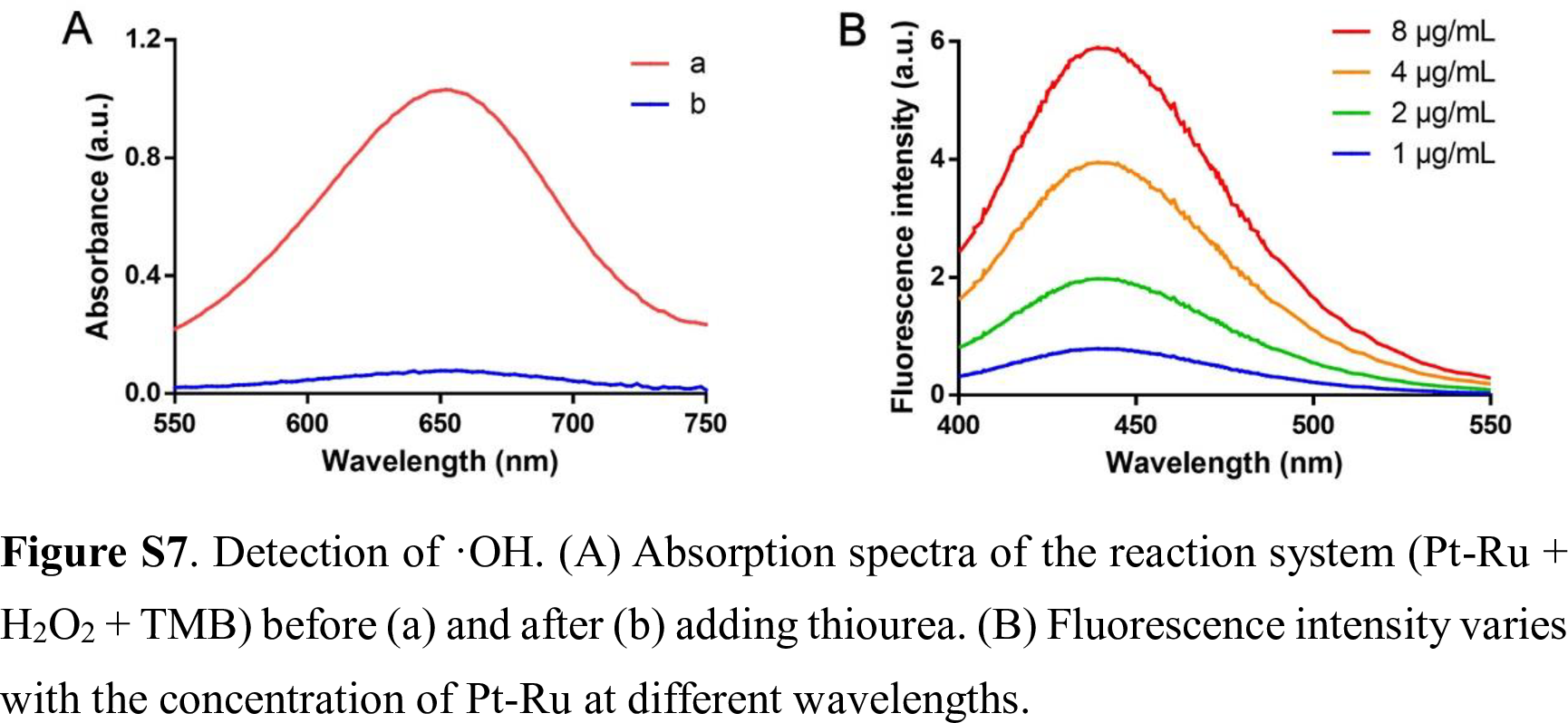

Supplement: S7 Fig — (TIF) [file pone.0301358.s007.tif]

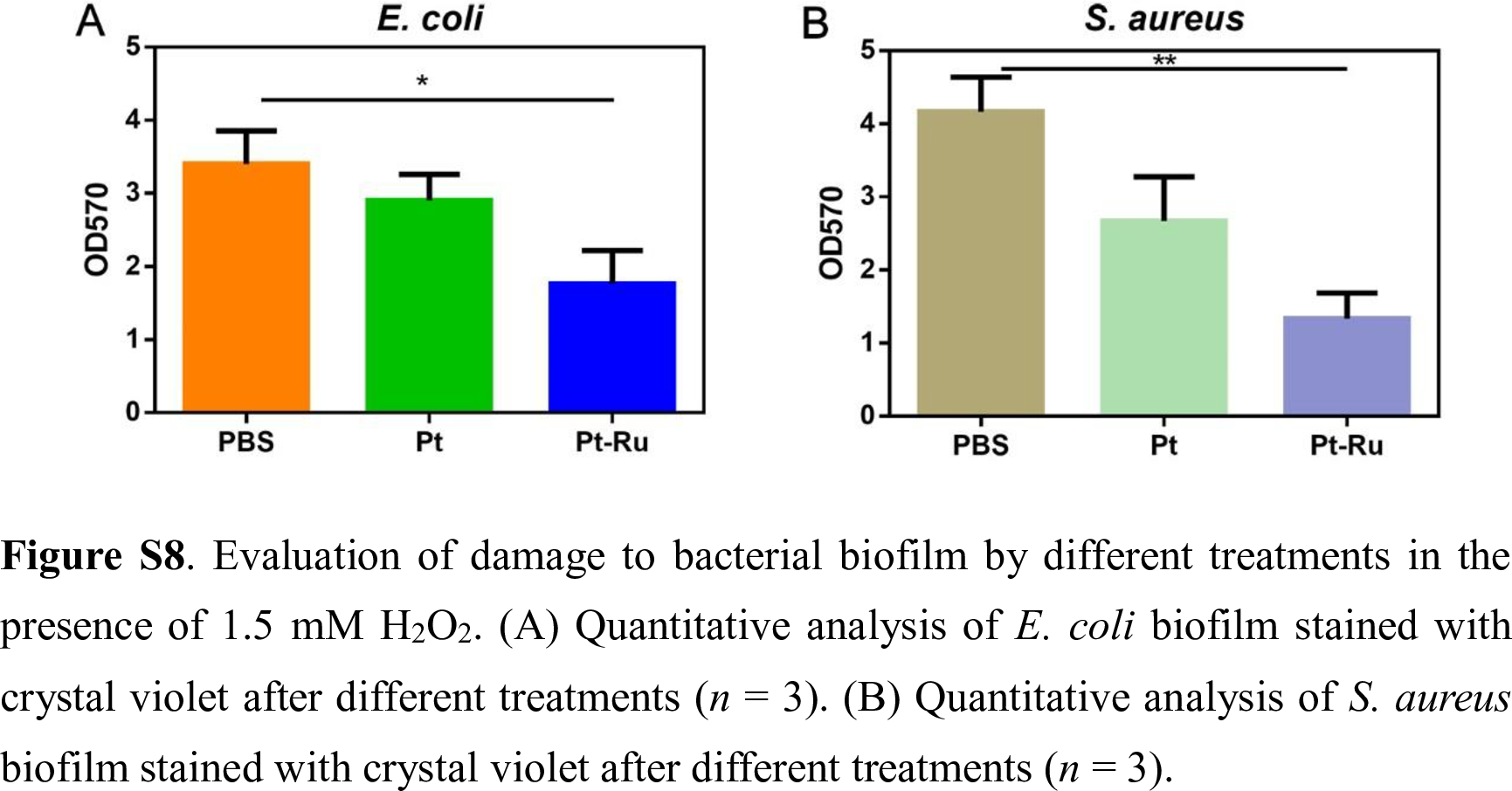

Supplement: S8 Fig — (TIF) [file pone.0301358.s008.tif]

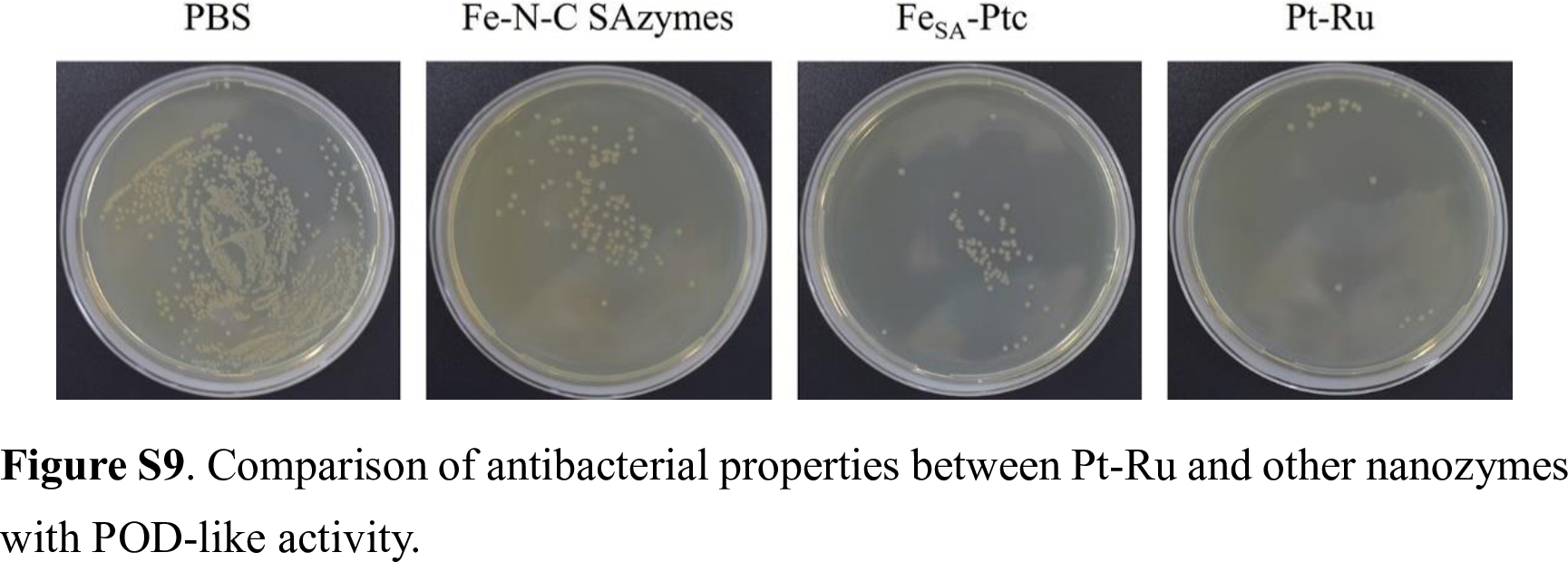

Supplement: S9 Fig — (TIF) [file pone.0301358.s009.tif]

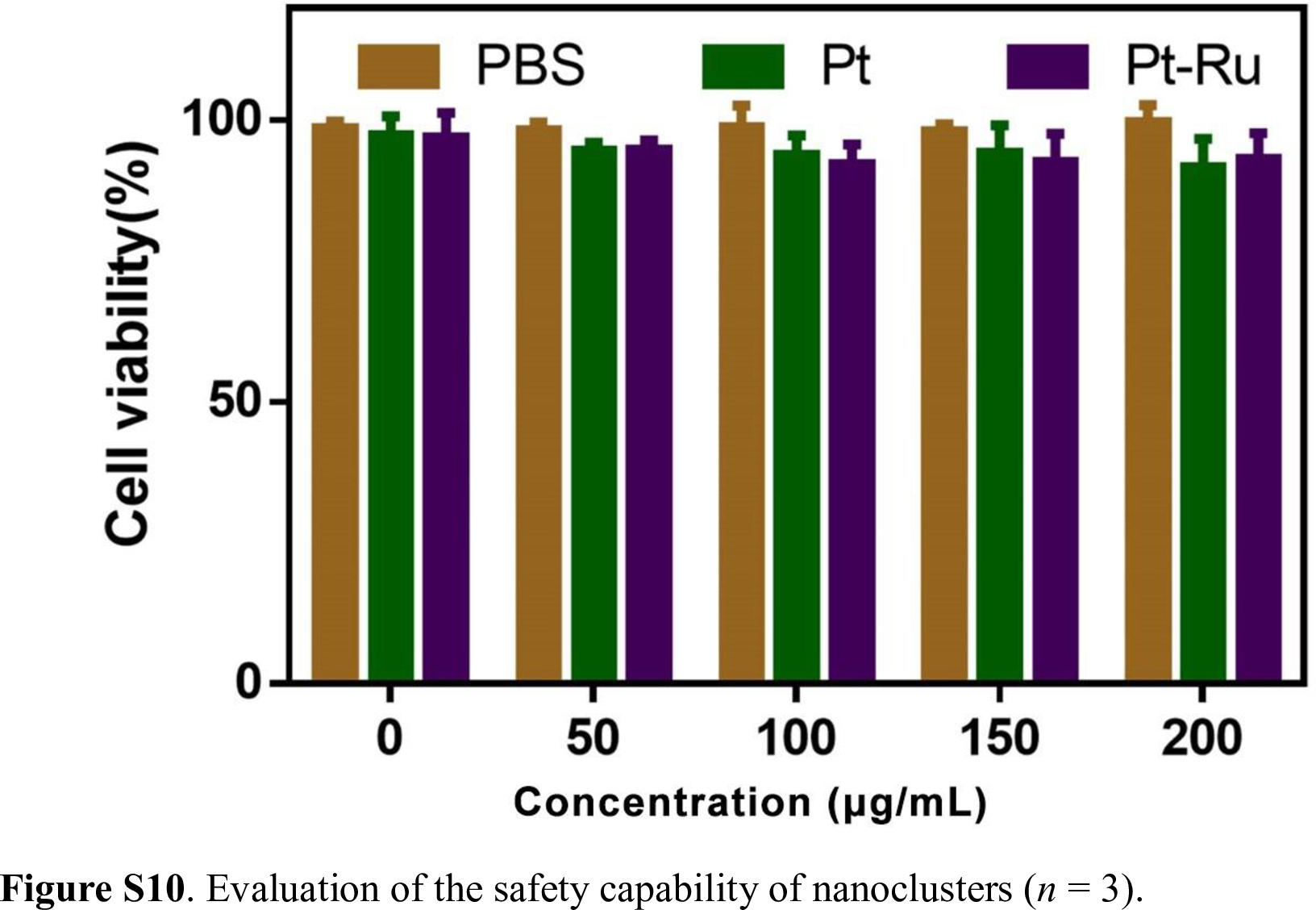

Supplement: S10 Fig — (TIF) [file pone.0301358.s010.tif]
